# Supplementary material for: A Bayesian approach to estimate the probability of resistance to bedaquiline in the presence of a genomic variant
Source: PLoS One. 2023 Jun 14;18(6):e0287019. doi: 10.1371/journal.pone.0287019 (PMC10266631; doi:10.1371/journal.pone.0287019)
Supplement: S1 Fig — (DOCX) [file pone.0287019.s001.docx]

**Figure S1:** Study selection after the published systematic review and individual meta-analysis
