# Supplementary material for: A Bayesian approach to estimate the probability of resistance to bedaquiline in the presence of a genomic variant
Source: PLoS One. 2023 Jun 14;18(6):e0287019. doi: 10.1371/journal.pone.0287019 (PMC10266631; doi:10.1371/journal.pone.0287019)
Supplement: S1 Table — (DOCX) [file pone.0287019.s002.docx]

**Table S1:** Prior probablity distribution parametrised by beta shape parameters (α and β) based on experts response in the three of genes of interest

| **Genes** | **Mutation class** | **n*** | $\hat{\boldsymbol{\alpha}}$ **^@^** | **SE(̂**$\hat{\boldsymbol{\alpha}}$**)** | $\hat{\boldsymbol{\beta}}$**^@^** | **SE(**$\hat{\boldsymbol{\beta}}$**)** | **Mean** | **Median** | **Variance** | **IQR** |
| --- | --- | --- | --- | --- | --- | --- | --- | --- | --- | --- |
| ***atpE*** | Synonymous mutation | 32 | 0.135 | 0.005 | 4.541 | 0.377 | 2.9% | 0.1% | 0.5 | 1.9% |
|  | Inframe indel | 31 | 0.607 | 0.023 | 0.793 | 0.032 | 43.4% | 39.6% | 10.2 | 58.7% |
|  | Missense mutation | 30 | 0.840 | 0.034 | 0.630 | 0.025 | 57.0% | 61.0% | 9.9 | 57.4% |
|  | Homoplastic mutation | 33 | 0.508 | 0.022 | 0.305 | 0.011 | 62.4% | 74.6% | 12.9 | 68.9% |
| **Rv0678** | Synonymous mutation | 32 | 0.078 | 0.003 | 0.315 | 0.016 | 19.8% | 0.2% | 11.4 | 24.0% |
|  | Nonsense mutation | 33 | 1.550 | 0.077 | 0.380 | 0.014 | 80.0% | 90.3% | 5.4 | 29.5% |
|  | Frameshift mutation | 32 | 1.550 | 0.076 | 0.480 | 0.018 | 76.0% | 85.2% | 5.9 | 34.6% |
|  | Inframe indel | 30 | 1.112 | 0.042 | 1.257 | 0.053 | 46.9% | 456.0% | 7.4 | 45.6% |
|  | Missense mutation | 30 | 1.596 | 0.067 | 0.932 | 0.037 | 63.1% | 67.0% | 6.6 | 41.4% |
|  | Homoplastic mutation | 33 | 0.904 | 0.039 | 0.476 | 0.018 | 65.5% | 74.0% | 9.5 | 52.9% |
| ***pepQ^#^*** | Synonymous mutation | 10 | 0.027 | 0.001 | 0.584 | 0.066 | 4.4% | 26.0% | 2.6 | 26.1% |
|  | Nonsense mutation | 10 | 0.548 | 0.022 | 0.430 | 0.017 | 56.0% | 47.9% | 12.4 | 51.1% |
|  | Frameshift mutation | 10 | 0.543 | 0.021 | 0.476 | 0.019 | 53.3% | 46.1% | 12.3 | 51.0% |
|  | Inframe indel | 9 | 0.406 | 0.015 | 0.954 | 0.040 | 29.9% | 32.9% | 8.9 | 43.3% |
|  | Missense mutation | 10 | 1.499 | 0.055 | 2.680 | 0.113 | 35.8% | 38.0% | 4.4 | 37.5% |
|  | Homoplastic mutation | 10 | 0.598 | 0.024 | 0.460 | 0.018 | 56.6% | 48.2% | 11.9 | 50.2% |

**n*-**the number of responses varied depending on the belief of the expert whether the gene plays a role in BDQ resistance and the response for a specific type of variant

**#** The mean and the variance is estimated from experts that believe *pepQ* plays a role in BDQ resistance (one of the components in the mixture distribution) and therefore the result presented here is not the final one.

**^@^** Two-thirds of the experts assumed that the effect of mutations on the phenotype that occurs in laboratory experiments can be extrapolated to the phenotype of clinical isolates containing the same variant. A single prior distribution was thus constructed combining the information from experts answering "Yes" (n1=22, p(X1)=0.667) and reflecting the uncertainty coming from experts answering the question about extrapolation as "I don't know" or "No" (n=11, p(X2)=0.333)***.***

**NB:** For Rv1979c the mean prior probablity is not presented in this table because all experts respond “No” or “I don’t know” for the survey question “can mutations in gene Rv1979c confer resistance to BDQ”.
