# Supplementary material for: A Bayesian approach to estimate the probability of resistance to bedaquiline in the presence of a genomic variant
Source: PLoS One. 2023 Jun 14;18(6):e0287019. doi: 10.1371/journal.pone.0287019 (PMC10266631; doi:10.1371/journal.pone.0287019)
Supplement: S2 Table — (DOCX) [file pone.0287019.s003.docx]

**Table S2:** Mixture distribution and mixing proportion for the prior distribution of *pepQ* and Rv1979c

| **Gene** | **Mutation** | **Mixture distribution #** | **Prior median** | **IQR** |
| --- | --- | --- | --- | --- |
| pepQ  *(n=33,*  *yes=12,*  *no=4,*  *I do not know=17)* | S**ynonymous** | f(x)= 0.36*beta(0.027, 0.584) + 0.12*I(x=0) + 0.52*beta (1, 1) | 26.0% | 26.1% |
|  | Nonsense | f(x)= 0.36*beta (0.548, 0.43) + 0.12*I(x=0) + 0.52*beta (1, 1) | 47.9% | 51.1% |
|  | Frameshift | f(x)= 0.36*beta(0.543, 0.475) + 0.12*I(x=0) + 0.52*beta (1, 1) | 46.1% | 51.0% |
|  | Inframe indel | f(x)= 0.36*beta(0.406, 0.954) + 0.12*I(x=0) + 0.52*beta (1, 1) | 32.9% | 43.3% |
|  | Missense | f(x)= 0.36*beta(1.499, 2.688) + 0.12*I(x=0) + 0.52*beta (1, 1) | 38.0% | 37.5% |
|  | Homoplastic | f(x)= 0.36*beta(0.598, 0.460) + 0.12*I(x=0) + 0.52*beta (1, 1) | 48.2% | 50.2% |
| Rv1979c  *(n=32,*  *yes=2,*  *no=5,*  *I don’t know =25)* | S**ynonymous** | f(x)= 0.16*p(x=0) + 0.84*beta (1, 1) | 42.0% | 42.0% |
|  | Nonsense | f(x)= 0.16*p(x=0) + 0.84*beta (1, 1) | 42.0% | 42.0% |
|  | Frameshift | f(x)= 0.16*p(x=0) + 0.84*beta (1, 1) | 42.0% | 42.0% |
|  | Inframe indel | f(x)= 0.16*p(x=0) + 0.84*beta (1, 1) | 42.0% | 42.0% |
|  | Missense | f(x)= 0.16*p(x=0) + 0.84*beta (1, 1) | 42.0% | 42.0% |
|  | Homoplastic | f(x)= 0.16*p(x=0) + 0.84*beta (1, 1) | 42.0% | 42.0% |

**#** The prior distributions for *pepQ* were estimated from three components with a different mixing proportion; The mixture proportions of each component were set equal to the observed probability of responding yes, no or I do not know. “yes” (n1 = 12; $w_{1}=0.36$), “no” (n2 = 4; $w_{2}=0.12$), or “I do not know” (n3 = 17; $w_{3}=0.52$ ) out of the total number included experts (n=33). Whereas for Rv1979c The observed empirical probability of responding “no” (n1 = 5; $w_{1}=0.16$), “I do not know” (n2 = 27; $w_{2}=0.84$), out of the total number included experts (n=32). Two experts who responded "yes" were included in the group who answered "I do not know" because they also disagreed.

**I(.)** is the indicator function being one if the argument is true

IQR: Interquartile range
